# South African Foundation Phase Teachers’ Perceptions of ADHD at Private and Public Schools

**DOI:** 10.3390/ijerph120303042

**Published:** 2015-03-11

**Authors:** Anwynne Kern, Zaytoon Amod, Joseph Seabi, Adri Vorster

**Affiliations:** School of Human and Community Development, University of the Witwatersrand, Private Bag 3, WITS, Gauteng 2050, South Africa; E-Mails: zaytoon.amod@wits.ac.za (Z.A.); joseph.seabi@wits.ac.za (J.S.); adri.vorster@wits.ac.za (A.V.)

**Keywords:** Attention Deficit Hyperactivity Disorder (ADHD), teacher perceptions, incidence, aetiology and rates

## Abstract

This study investigated foundation phase teachers’ perceptions of Attention Deficit Hyperactivity Disorder (ADHD). The teachers’ views on the aetiology, appropriate interventions and incidence rates of ADHD were examined. A total of 130 foundation phase teachers from mainstream private and public schools completed a self-developed questionnaire that had been piloted by the researchers. Both descriptive and inferential statistics were used to analyse the data, specifically to determine whether there were differences in responses between public and private school teachers. Thematic content analysis was used to identify the themes that emerged from the open-ended questions. It was found that the teachers had a limited understanding of ADHD, in terms of what it is as well as the aetiology. In addition, it emerged that medication was the preferred method of intervention despite the participants’ awareness of alternative intervention methods. A comparison of the private and public school teachers’ results indicated no significant difference in their perceptions regarding the aetiology, interventions or incidence rates of ADHD.

## 1. Introduction

As one of the most publicized conditions affecting children over the past two decades [1], there is an increase in ADHD-related behaviour in our classrooms [2]. Researchers agree that ADHD is diagnosed in approximately 3%–10% of children internationally [3,4], with the ADHD support group of South Africa placing the incidence rate at 10% [5]. These statistics make ADHD one of the most frequent reasons for referral to school psychologists [1,5,6,7,8] and child psychiatric facilities [9]. The ADHD symptoms of inattention, impulsivity and hyperactivity become more evident in the classroom, positioning teachers in a unique place to identify and refer these learners for further assessment [9,10]. Despite this reliance on teachers by parents, studies have found that teachers can provide inaccurate and inappropriate advice to parents [11]. This highlights the need to examine what teachers know about ADHD as they play an important part in its identification and intervention planning.

While there are numerous studies on teachers’ perceptions of ADHD it is important to note that there are a number of aspects that influence perception, such as stigma, experience, knowledge and prior learning. The current study focused primarily on teachers’ understanding and knowledge of ADHD. There is no documented research on private and public school teachers’ perceptions of ADHD in South Africa. 

A number of studies have reported that teachers’ perceptions of the incidence of ADHD in their classrooms are considerably higher than the existent prevalent rate of this condition amongst children [1,12,13,14,15]. This implies that children could be ‘identified’ as having ADHD, when in fact there may be other factors impacting on their attention or activity levels. It also brings into question teachers’ ability to accurately identify learners who may have ADHD, as opposed to those who are merely hyperactive or inattentive because of other factors.

Holz and Lessing ([16], p. 238) had identified the problem that faces teachers today and they noted that “teachers … are generally not trained to identify or teach learners with ADHD.” This appears to also be true in the South African context. The Education White Paper 6 promotes the inclusion of all learners into mainstream classes. It asserts that classroom teachers will be the primary resource for achieving the goal of inclusive education. This means that teachers will need to acquire new skills as well as improve their existing skills and knowledge [17]. However pre-service teacher training programmes do not generally provide them with the tools to successfully implement inclusive education, and to identify and address the needs of learners presenting with ADHD. In addition research has found that knowledge regarding ADHD increases with increased exposure to children diagnosed with ADHD [18]. Thus while pre-service training creates an awareness of ADHD, theoretical exposure alone does not necessarily enhance an individual’s knowledge of ADHD. While teachers are not qualified to diagnose ADHD, they are ideally placed to identify learners who may have the disorder in order to refer them for further assessment. This is particularly the case as the symptoms of hyperactivity and inattention related to ADHD are usually noticed when children start attending formal schooling due to the structure of this environment. It can be challenging for children who were previously in pre-school or at home, and consequently a less structured and demanding environment, to meet the behavioural and sometimes academic demands of the classroom [4,19]. The inattention and hyperactivity, characteristic of ADHD therefore only seems to appear when the children are sitting in front of a teacher who expects a certain level of attention and the ability to remain seated.

This study examined South African foundation phase teachers’ perceptions surrounding ADHD since the symptoms of ADHD usually have their onset during this phase of children’s schooling Teachers’ thoughts on the incidence rate, aetiology and possible interventions for children presenting with ADHD were explored. While international studies have explored similar aspects of ADHD it has been demonstrated that culture plays a role in the individuals’ perception of ADHD [18]. Consequently results from international studies cannot necessarily be generalised to the South African context. However, it can be argued that culture does not merely relate to ethnicity. In South Africa the culture within private and public schools does differ. This difference lies not only in the race of children attending these schools, but also the socio-economic status of the learners, and by association the teachers since they will have greater resources available to them in terms of access to training, teaching material and so forth. Children in public schools are more diverse given that it serves a large community of people creating a different school culture from private schools which are predominantly attended by English first language speakers from high socio-economic status groups. Based on these cultural differences as well as the differences in resources available, the researchers examined if there would be a difference in the perceptions of ADHD amongst a sample of foundation phase teachers in mainstream public and private primary schools in the Gauteng province of South Africa.

The essential futures of ADHD as stated in the DSM-5 points to a pattern of inattention, impulsivity and hyperactivity which is persistent and present in two or more settings [20]. In relation to the school setting, teacher knowledge regarding ADHD is important as he or she is usually the first person to notice the inattention and hyperactivity due to the structured nature of the school environment [4,21]. Furthermore, the symptoms of ADHD characteristically worsen in situations that necessitate sustained attention or mental effort or the lack of intrinsic appeal or novelty. Consequently foundation phase teachers are usually the first to identify the disorder in children. However, despite the necessity for factual teacher knowledge regarding ADHD, a study conducted in public schools in the Western Cape province of South Africa found that teachers lacked knowledge as regards ADHD [5]. This is consistent with international research [10] that established that teachers had significant gaps in their knowledge regarding, aetiology, symptomatology and treatment of ADHD. This lack of knowledge can result in teachers giving both inappropriate and incorrect advice to parents, who often view teachers as more knowledgeable than themselves. The teachers’ approach to learners is also altered based on their often incorrect perceptions, causing them to stigmatize the learner diagnosed with ADHD [4,22]. Understanding ADHD, in the South African context of inclusive education, will allow the teacher to scaffold the curriculum accordingly and adapt the teaching style used to make the curriculum accessible to all learners, specifically those diagnosed with ADHD. 

ADHD is divided into three subtypes according to the main features of each type, namely ADHD: Predominantly Inattentive Type; ADHD: Predominantly Hyperactive-Impulsive Type; and ADHD: Combined type. Kern and Seabi [14] found that teachers only referred to the behavioural component of ADHD which means that the teachers focused on the externalized behaviour [22] highlighting the symptom of inattention and hyperactivity of children with ADHD. They did not consider the essential feature of impulsivity, the age of onset, number of settings the behaviour was evident in, as well as co-morbid disorders associated with ADHD. As a result teachers were less likely to recognize the inattentive type of ADHD as was also found by Moldavsky, Pass and Salyal [23].

According to Mowbray ([24], p. 13) “the condition is thought to be triggered by the interaction between the child’s biology and the environment”. This viewpoint is supported by Faroane [25] and Sadock and Sadock [26] who alluded that ADHD is a multifactorial disorder, perpetuated by the additive effects of genes and environmental risk factors including emotional disturbance and stressful life events. Within South Africa this becomes especially relevant when one considers that environmental factors, such as lead poisoning, poverty and inadequate living conditions can affect the development of the disorder [19]. Parenting style, qualities of early attachment, presence of parental and sibling physical or mental illness and social and cultural influences, have all been implicated in the development of the disorder [15,27]. Vorster [28] highlights how repeated trauma, parenting styles and child-parent interactions either maintain or exacerbate the course of the disorder. On the other hand, Perold, Louw and Kleynhans [5] view parenting style as a possible protective factor which can moderate the effects of ADHD as opposed to causing it. The aforementioned causes of ADHD are however not subscribed to by many teachers. Instead the predominant causes mentioned by teachers are diet and parenting style (5, 10), which researchers agree may exacerbate the symptoms, but neither of which cause the disorder [24,29]. 

One cannot negate the biological cause of ADHD, however the environment and various interactions with others does affect the manner in which ADHD is displayed. So while the disorder and etiology thereof is a distinct entity, a number of other factors exacerbate or mimic the symptoms of ADHD. The stress resulting from the excessive demands of modern day schooling, unemployment, marital problems, substance abuse, general abuse and neglect from parents causes children to display the symptom of inattention in ADHD [24,26]. Consequently, if one examines the school and home system one may find that the child’s behaviour is symptomatic of a disorder in the school or home situation, and not a neurological disorder [1].

In the South African context it is important to consider both intrinsic and extrinsic factors in the development of ADHD. Some of the most prominent extrinsic factors include social-economic barriers, inflexible curricula, inappropriate and inadequate provision of support services and lack of parental recognition and involvement. Language and communication difficulties, health difficulties, sensory impairments and intellectual and learning difficulties are some of the most prominent intrinsic factors [29].

Researchers have found a number of causes for the appearance of attention difficulties. A child who is hearing impaired may appear to be dreamy and lacking concentration [30]. This child may also be talkative, disruptive and display gaps in learning. A lack of visual skills may result in poor work, difficulty reading, paying attention or even behavioural problems. Inattention and distraction are often symptoms displayed by children with reading disorders [31]. Some children are oversensitive to sight, sounds and smells and will experience a sensory overload in a busy classroom, which distracts them and make them appear inattentive [32]. Additionally, Kantrowitz and Springen [33] have found growing evidence that a chronic lack of sleep can mimic the symptoms of an attention deficit disorder. Sadock and Sadock [26] suggest that although food additives, colourings, preservatives, and sugar may cause hyperactive behaviour, no scientific evidence denotes that these factors cause ADHD. Temperament is another factor that should be considered a predisposing factor to ADHD [26]. While temperament at the extreme end of the continuum may be more arduous for parents and teachers to manage, these behaviours are still considered normal [34].

While no quick treatment exists for ADHD, behaviour can be managed through the use of an educational programme that fits the child’s specific needs and medication, if the parents and doctors think that it will be beneficial [35]. DuPual and White [36] specify three intervention methods. These are:

▪ Medical Interventions which are central nervous system stimulants.
▪ Behavioural Interventions, and
▪ Academic Interventions.

Medical interventions usually involve the use of central nervous system stimulants. Research into the efficacy of stimulant medication found that between 70% and 90% of children treated with medication responded positively while the remainder of the children displayed no response or the ADHD symptom worsened [37,38]. Non-stimulant medications are also used in the treatment of ADHD [39]. Conflicting evidence exists on teachers’ views on the use of medication. Some researchers have found that teachers oppose the use of medication in the treatment of ADHD and feel that it is overused [10]. In fact some teachers expressed confidence in their ability to deal with children diagnosed with ADHD and felt that their educational interventions were effective [23]. However Glass and Wegar [1], and found that the teachers in their study favoured stimulant medication as a treatment option. 

Behavioural interventions for ADHD are divided into two distinct categories. The first category refers to changing antecedent events which focus on changing behaviour prior to the specific behavior requiring change. Examples of these interventions include posting rules, modifying assignments and peer tutoring. This is supported by Grandy and McLaughlin [40] who refers to antecedent conditions as those relating specifically to the setting and environmental conditions. One of the areas that they focus on is that of seating. Placing children with ADHD near to the educator so that they can receive additional support from the educator is one suggestion.

The second behavioural intervention uses the cognitive-behavioural approach. This refers to consequent events which employ both positive and negative consequences for a specific behaviour. Examples include star charts, time out and privileges. These interventions specifically focus on the child’s thinking processes and aim to encourage children to problem-solve using an appropriate strategy while simultaneously weighing up the consequences of their actions. 

Academic interventions entail the offering of academic support to learners diagnosed with ADHD. Examples include peer tutoring and individualized direct instruction [36]. Previous research has shown that peer tutoring can result in improved “on-task behaviour, activity level and academic performance” in learners suffering from ADHD ([40], p. 65). This intervention was found to be preferable by teachers in the UK who expressed feeling of competence in dealing with children experiencing difficulties [23]. 

On the other hand, Evans, Schultz and Sadler [41] included the importance of social interventions in their study. They argued that peer rejection and aggression towards peers is predicative of a range of serious adjustment problems in children with ADHD. However, despite acknowledging the importance of implementing social skills training, the training itself was not effective. This outcome was possibly due to the children’s inability to generalize the skills learnt in treatment to other settings. 

Despite having knowledge of the various intervention methods available, studies show that teachers prefer medication as an intervention strategy since they regard it to be more effective and timely [6,14]. Glass and Wegar [1] also found that many teachers believe that medication is warranted for the control of the behaviours that are characteristic of ADHD, even when the educator believes that ADHD is not a biological condition and that it is one that is caused by environmental factors. It is believed that teachers’ preference for medication aims to alleviate their own feelings of frustration, confusion and incompetence with regards to children exhibiting ADHD [16].

It is evident from the literature that ADHD is prominent in all communities and cultures. While the cause is influenced by biological, psychological as well as social systems, the prevalent interventions do not necessarily address these various systems. The understanding of both the causes and interventions are informed by the model from which ADHD is understood. 

While there are a number of distinct models upon which an understanding of ADHD can be based, research shows that teachers are more prone to favouring the medical model to address the symptoms and favour medication to alleviate the symptoms of ADHD due to the fast acting nature of the medication [1]. Mowbray ([24], p.12) asserts that “teachers think of these children as the “me generation” who cannot cope with anything other than instant gratification, who have led disrupted and disheveled early lives dominated by too much television”. As a result children struggle to exercise self control for extended periods of attention. In fact teachers have complained about having to “perform” or “act” in front of their classes just to keep the children’s attention. They argue that children are accustomed to seeing moving pictures and so cannot attend to someone who is standing still. These children are often labeled with ADHD by their teachers. 

In contrast to the medical model, a broader socio-ecological model, such as the eco-systemic model acknowledges that interactions between an individual and various systems may either hinder or augment their development [42,43]. Applied to ADHD the eco-systemic perspective helps us to understand that children cannot be viewed in isolation, but as part of the bigger whole and in a reciprocal relationship with it [5]. The eco-systemic model therefore takes into consideration that inattention can also be attributed to environmental factors that negatively affect the learner, such as teaching style, classroom noise, parental style and socio-economic hardships. Given that the school environment forms part of a child’s “bigger whole” it is important to investigate one aspect of this environment, that is the teacher, and their attitudes and perceptions around ADHD as this will ultimately inform the interaction between the teacher and the child. 

The present study aimed to (a) establish what teachers understood about ADHD, (b) establish the perceived and actual incidence rate of ADHD, (c) identify what teachers perceived to be the aetiology of ADHD and (d) ascertain teachers’ perceptions on the possible interventions for learners presenting with ADHD. Both private and public school teachers’ views in relation to the above research questions were explored.

## 2. Experimental Section

### 2.1. Context of the Study

The present research study was conducted at fifteen mainstream primary schools in the Johannesburg East, West and North Districts of the Gauteng region of South Africa. Amongst these, eight were private and seven were public schools. Of the total number of participants, 84% (*n* = 106) indicated that their schools were located within an urban setting, while 8% (*n* = 10) of the respondents, reported that their schools were either township schools or inner city schools. In South Africa, a township refers to an under-developed urban living area on the outskirts of towns or cities, primarily reserved for people of colour during the apartheid era. While the highest number of teachers, (*n* = 56), indicated that they had access to educational psychologists, the public school teachers (*n* = 38), indicated more access to educational psychologists in comparison to their private school counterparts (*n* = 18). This is an unexpected finding and is unique to this study as private schools are better funded and resourced. Thirteen teachers indicated that they had access to clinical psychologists.

### 2.2. Research Design

The study employed a non-experimental, descriptive design in that it aimed to explore teachers’ perceptions of ADHD. 

#### 2.2.1. Sampling Procedures

Of the twenty-seven primary schools who were invited to participate in the study, only fifteen principals gave consent for their teachers to participate. The sample consisted of 130 foundation phase teachers from fifteen schools who indicated that they would participate in the study. The sample was a purposive sample of convenience. Foundation phase teachers were specifically selected since they come into contact with learners between the ages of six and nine and it is during this time that the symptoms of ADHD are usually first recognized as potentially problematic in children [36]. 

#### 2.2.2. Participants

The participants in the current study comprised of 129 females and one male whose ages ranged between 20 and over 50 years. The majority of the participants were aged over 50 years (26%, *n* = 34), while the least number of participants, (8%, *n* = 10), were within the youngest age range, 20–25 years. The overall mean age of the sample was 36–40 years of age. 

Of the 130 participants, 50 (38%) were employed at private schools, while 80 (62%) were employed at public schools. Twenty-five participants (19%) reported that they were completing their postgraduate studies. Fifty-four percent (*n* = 59**)** of the participants indicated that they had not attended any training course on ADHD while, 46% (*n* = 70) indicated that they had undergone in-service training relating to ADHD. 

#### 2.2.3. Testing and Intervention Procedure

After ethical approval was obtained for this study from the University of the Witwatersrand’s Human Research Ethics Committee (Non-Medical), written consent was obtained from the Gauteng Department of Education and from the principals of the fifteen schools via individual meetings with the first author. Teachers were requested, in writing to participate in the study. They were informed, through the use of an information sheet that participation in the study was completely voluntary and that anonymity and confidentiality would be ensured. No identifying information was requested on the questionnaire.

#### 2.2.4. Data Analysis

Both qualitative and quantitative data was gathered. The quantitative data was analysed using descriptive statistics employing frequency analysis, based on the aims and research questions of the study. A *t*-test analysis was used to ascertain the differences in responses between the public and private school teachers’ perceptions of ADHD. Having been coded and entered into a spreadsheet, the quantitative data was analysed Statistical Analysis Software (SAS).

The open-ended questions were analyzed using thematic content analysis which consists of “burrowing through written records in order to discover their characteristics” ([44], p. 81). Braun and Clarke [45] distinguish between two types of thematic content analysis, namely, an inductive thematic analysis and theoretical thematic analysis (deductive). For the present study, a theoretical analysis was used. This is an approach wherein the researcher fits the data into a pre-existing coding frame [45]. Theoretical thematic analysis also known as deductive category analysis works with aspects of analysis which have been formulated prior to data collection [46]. In this study the categories for analysis were based on the aims and research questions of the study, while the themes were based on the pattern of responses that emerged. While the categories of analysis are deductive categories, the patterns of responses were then analyzed employing frequency analysis. 

### 2.3. Instrumentation

A number of international studies which investigated teachers’ perceptions of ADHD have used self-developed instruments [10,18,23]. The current study also used a self-developed questionnaire (Supplementary A) which was designed based on relevant ADHD literature.

This self-developed questionnaire (Supplementary A) demonstrated adequate internal consistency (Cronbach’s α = 0.912). Face and content validity of the questionnaire was assessed through the piloting process. The questionnaire was piloted on foundation phase educators who did not form part of the sample of the study, to evaluate the clarity of the specific questionnaire items [47]. The pilot sample consisted of five, female educators. Two of the educators were teaching Grade 2, two were teaching Grade 1, while one of the educators was teaching Grade 3. The results of the pilot study indicated that the questionnaire items were clearly understood, requiring no modification. 

The questionnaire was two-fold in nature, consisting of both open and closed-ended questions, with the knowledge and attitudes about ADHD being evaluated through the use of closed-ended questions employing a Likert scale ranging from Strongly Disagree to Strongly Agree. However the results have been analyzed and interpreted using a three point scale; Agree, Neither Agree or Disagree and Disagree; where the Strongly Agree and Agree results have been combined, as well as the Strongly Disagree and Disagree results. This was done to facilitate the presentation and interpretation of the results. 

Questions 1 to 13 explored the demographic background of the participants and were used in exploring the similarities and differences between private and public schools teachers’ perceptions of ADHD. This section included amongst others, questions around gender, age, qualifications, teaching experience, type of school and number of learners in a class. Questions 14 and 15 examined both the actual and perceived incidence rates of ADHD in the teachers’ respective classrooms along with exploring the actual treatment plans for children diagnosed with ADHD as well as the teachers’ thoughts on the efficacy of various treatment options. Questions such as *“Do you have learners who have been medically diagnosed as having ADHD in your class?”*, and *“What is their treatment plan?”*, were presented. Question 16 asked *“what is your understanding of the term Attention Deficit Hyperactivity Disorder?”* aimed at gauging teachers’ overall understanding of ADHD. Questions 17–18 were used to evaluate the teachers’ knowledge regarding the diagnostic criteria for ADHD and were based on the diagnostic criteria specified in the DSM-IV as the study was conducted prior to the publication of the DSM-5. Questions 19–22 evaluated the teachers’ perceptions regarding the causes and interventions for ADHD. Question 19 and 21 were formulated based on the literature presented on perceived and actual causes and interventions for ADHD. 

## 3. Results and Discussion

### 3.1. Understanding of ADHD

The results of the study indicated that the teachers’ understanding of ADHD focused on the type of behaviour that the child exhibited. Specifically, the behaviours highlighted by the teachers were an inability to sit still, remain focused, complete work and sustain concentration. While these features are not considered diagnostic they are highlighted by Sadock and Sadock [26] and O’Neil [38] as distinguishing features of ADHD. The emphasis placed on the ability to sit still was higher in public school teachers (15%, *n* = 12) than in private school teachers (10%, *n* = 5). This may be linked to the class size where the mean class size of the public school teachers were 30–34 learners per class in comparison to the private school teachers where the mean class size was 21–25 learners. 

While the neurology of ADHD has been emphasized by numerous authors [1,24,25,48], only 15% (*n* = 16) of all of the teachers alluded to the fact that ADHD is a neurological condition. Twenty percent (*n* = 10) of the private school teachers referred to the neurology of the condition in comparison to the 8% (*n* = 6) of public school teachers. The discrepancy between the knowledge of private and public school teachers may be as a result of their course attendance, where 55% (*n* = 28) of private school, and 40% (*n* = 32) of public school teachers attended courses related to ADHD. The result seems to indicate that private school teachers have a better understanding of ADHD, possibly due to them attending ADHD courses, in comparison to their public school counterparts.

**Figure 1:**
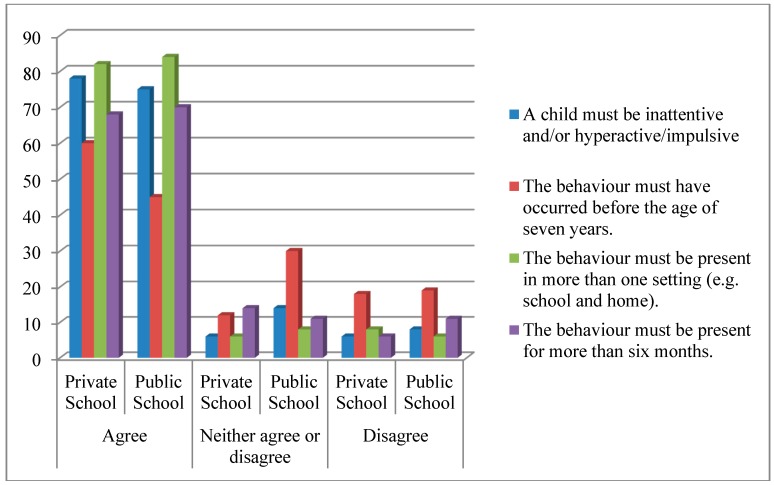
Comparison regarding the diagnostic criteria of ADHD.

Figure 1 presents the comparison of private and public school educators’ opinions regarding the diagnostic criteria for ADHD. Seventy-seven percent (*n* = 100) of the teachers agreed that a child must be inattentive and or hyperactive/impulsive for a diagnosis of ADHD to be made. This is consistent with Amod, Vorster and Lazarus’ [4] findings. This trend was also noted in a study involving Chinese teachers [18]. 

However, a different picture emerges with reference to the criterion that states that the behaviour must have occurred before the age of 7 years. Overall, only 51% (*n* = 66) of the teachers agree with this criterion, where 60% (*n* = 30) of the private school teachers and 45% (*n* = 36) of the public school teachers showed an agreement with this criterion. Again course or workshop attendance (45% of public school and 55% of private school teachers) may account for the variability in their responses.

### 3.2. Incidence and Ratios

The overall reported diagnosed incidence rate of ADHD was 5% which is within the research norms of between 3% and 10% [1,26]. The diagnosed incidence rate for ADHD was within the lower limits for the public school teachers (4%), while it was 7% for the private school teachers. There may be a link between the private school class size, 21–25 learners, and the slightly higher incidence rate. Parents may purposefully be seeking out private schools with smaller class sizes to ensure that their children receive more attention due to their special needs, which could account for the high incidence rate of ADHD at private schools. This finding is supported by Fabiano, *et al.* [48] who pointed out that pupil teacher ratio can have an impact on disruptive behaviours in a classroom. The results of the current study are in contrast to a study conducted by Seabi and Kern [12] in South Africa, where the perceived incidence rate was as high as 15%. This seems to indicate that the teachers in this study may be able to correctly screen learners with ADHD.

### 3.3. Perceived Causes of ADHD

Responses relating to the causes of ADHD can be classified into five themes as can be viewed in Table 1 below. These include physiological causes, emotional factors, diet, inadequate parenting and other barriers to learning, with the two highest reported causes being diet (72%, *n* = 94) and physiological factors (56%, *n* = 56). One of the respondents noted that ADHD is a *“neurologically based disorder associated with chemical imbalances in the brain affecting temperament and behaviour and performance at school”* which is *“usually inherited”*. These results are consistent with previous research which indicates that ADHD has a neurological basis [1,24,] and a resultant genetic link [49,50]. However, there was a discrepancy between the teachers’ understanding of the etiology of the disorder and the diagnostic criterion with only 12% (*n* = 16) of the teachers alluding to the neurological basis of ADHD with reference to the diagnostic criteria. 

Teachers seem to perceive poor diet as the primary cause of ADHD, with 76% (*n* = 38) of private school teachers and 70% (*n* = 56) of public school teachers indicating a positive response in this regard. While this result is consistent with teachers in America and Australia [10,19], it is in contrast to Sadock and Sadock [26] who state that there is no scientific evidence that indicates food additives, colourants, preservatives, and sugar may cause ADHD. They do however state that it may cause hyperactivity. 

Forty two percent (*n* = 54) of the teachers implicated barriers to learning as a causal factor while only 30% (*n* = 39) drew a link between emotional distress and the causality of ADHD, this despite emotional distress merely being a trigger to inattention and distractibility [51,52]. In this study 42 % (*n* = 55) of the teachers indicated that “inadequate” parenting might cause ADHD which is lower than the 80% of teachers in Sri Lanka who held a similar belief [13]. As relates to inadequate parenting there was a significant effect for television games in the current study, *t* = 0.250, *p* = 0.028 < 0.05, with public school teachers receiving higher scores than private school teachers.

**Table 1:** Comparative table of perceived causes.

Causes	Private School Educators						Public School Educators					
	Agree		Neither Agree nor Disagree		Disagree		Agree		Neither Agree nor Disagree		Disagree	
	*n*	%	*n*	%	*n*	%	*n*	%	*n*	%	*n*	%
Physiological	28	56	10	20	5	10	45	56	19	24	6	8
Emotional	13	26	13	26	17	34	26	33	25	31	18	23
Diet	38	76	4	8	6	12	56	70	7	8	9	11
Parenting	24	48	10	20	12	24	31	39	17	21	18	23
Other barriers to learning	26	52	7	14	13	26	46	55	16	20	11	14

### 3.4. Interventions for ADHD

As presented in Table 2 below, 52% (*n* = *68)* of the teachers regarded Ritalin as the most effective modality for treating ADHD. They indicated that *“children are able to achieve academically”*, it is effective *“almost immediately”*, and learners are able to *“focus better and complete tasks timeously”*. One of the teachers indicated that the children were *“motivated to work, join groups, finish work and could write on lines”*. 

**Figure 2:**
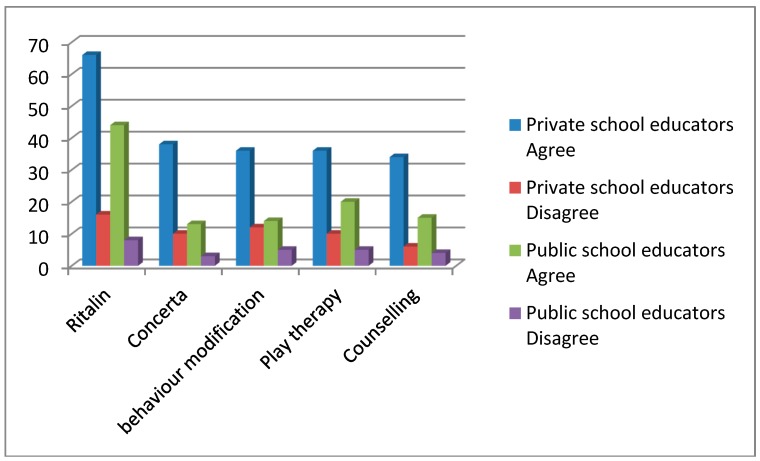
Comparisons of Private and Public School Educators’ Perceptions of the Efficacy of Various Treatment Modalities.

This preference for medication to treat ADHD is in contrast to the teachers’ belief that poor diet is the main cause of ADHD. This seems to corroborate the finding of previous studies [1,6], which suggested that teachers prefer medication as a way to control behaviours associated with ADHD. Despite this corroboration, it was found that teachers in a UK study felt differently towards medication and were in fact reluctant to endorse medication preferring instead to use educational interventions [23]. There is however a difference between public and private school teachers preference for medication with 66% (*n* = 33) of private school teachers and 44% (*n* = 35) of public school teachers indicating that they believe Ritalin to be an effective treatment for ADHD (see Figure 2). This descriptive difference may lie in the perception that parents and children from private schools have easier access to specialists who can diagnose and prescribe Ritalin. Seventeen percent of the teachers in this study were however not in favour of the use of medication. Some of their responses were, *“medication is not a cure for ADHD, it improves the key symptoms of inattention, hyperactivity and impulsiveness”* and *“in some children it seems to have very little effect”.* This correlates with Guerra and Brown’s [21] finding that teachers can be apprehensive about medication in the treatment of ADHD. 

**Table 2:** Educators’ Perceptions of the Efficacy of the Various Treatment Modalities.

Treatment Modalities	Agree		Disagree	
	*n*	%	*n*	%
Ritalin	68	52	14	11
Concerta	29	22	7	5
Behaviour modification	29	22	10	8
Play Therapy	34	26	9	7
Counselling	29	22	6	5

Twenty six percent (*n* = 34) of the teachers highlighted play therapy, and 22% (*n* = 29) counselling, as effective treatment modalities for ADHD. This, despite the finding that only 30% of the teachers specified that ADHD is caused by emotional distress. Eighty three percent of the teachers noted that a neurologist or an educational psychologist should assess children with ADHD. This high percentage of referrals to a neurologist is in contrast to the finding that only 56% of teachers reported that ADHD is in fact caused by neurological factors. This frequency of choice of a neurologist for referral was similar between private school teachers (86%) and public school teachers (81%). 

The intervention strategies chosen by teachers did not focus on a single strategy but instead on multiple interventions. They selected educator interventions, such as moving the child around the classroom, breaking assignments up into parts and ensuring that they have the attention of the child before giving instructions. An additional aspect of the educator interventions was that of behaviour modification. Ninety percent (*n* = 118) of the teachers employed a system of rewards which can be linked to Grandy and McLaughlin [40] behavioural intervention with a focus on consequent events. Parental interventions chosen by teachers in the current study included discussing the child’s diet with the parents; while medical interventions included requesting an assessment, and a medical examination to rule out any physical cause of the ADHD behaviour. The results indicate that teachers prefer medication as an intervention. However, despite having this preference for medication as an intervention strategy, 36% of the teachers did not expect a specialist to prescribe medication.

Based on the findings it appears that teachers’ understanding of ADHD is based primarily on the medical model as their focus appears to be on the child and not on other systemic factors that may result in the ADHD behavior. However their perceptions regarding the etiology of ADHD takes on a more systemic view as, factors such as parental style, and diet are alluded to. With regards to appropriate interventions, again a more systemic view was adopted by the teachers who amongst other interventions, attempted to adjust the environment to suit the particular child’s learning needs.

### 3.5. Limitations of the Study and Implications of the Findings

Several limitations of this study need to be acknowledged. First, given the small convenience sample size which mainly consisted of females, the results cannot be generalized to the wider teaching population. Moreover male participants may have held different perceptions regarding the various aspects of ADHD investigated. Furthermore, only schools in the Johannesburg East, West and North Districts were targeted which would also impact on the external validity of the results. In addition a 5-point Likert scale was used as opposed to a 4-point scale which may have resulted in a greater number of neutral responses. 

### 3.6. Implications for Future Research

The results of this study imply that teachers do not make a distinction between inattention and ADHD. This means inattentiveness, possibly caused by other factors, is identified by teachers as ADHD without any of the other diagnostic criteria being applied. Therefore, their understanding of the cause of ADHD includes physiological, dietary, parental, emotional and other barriers to learning as factors that may cause the disorder. Consequently, teacher training programmes, both pre- and in-service, on ADHD should include a focus on the causes of the disorder. Moreover, teachers need to be adequately trained to correctly screen learners with ADHD and implement the necessary intervention strategies to assist their learners. The identification process needs to address the possible causes of inattention and hyperactivity in children, over and above ADHD. These may include systemic factors such as emotional distress resulting from abuse or neglect, health difficulties, language difficulties and poverty. This training could be provided through collaboration between government departments and health practitioners, such as neurologists, pediatricians and psychologists, and could take on the form of pamphlets, educational talks and workshops, as well as personal communication. Given the range of systemic factors that could be implicated in the onset of the ADHD behaviours, particularly in the South African context, providing teachers with training to assist these learners is also paramount, both to the successful implementation of inclusive education, as well as the integration and education of these learners in positive learning environments. Moldavsky and Sayal [15] found that workshops and pamphlets aimed at improving knowledge regarding ADHD improved teachers’ approaches towards children with ADHD, their classroom strategies as well as their apparent ability to work with other individuals; such as parents and medical professionals, involved in the care of the child. 

Studies have found a correlation between teacher knowledge and student exposure [11]. This implies that teachers’ knowledge about ADHD increased with the increased exposure to children with ADHD. This is an area that is under researched in South Africa and has implications for new graduate teachers given that they would have had limited contact with learners with ADHD. 

## 4. Conclusions

Given the findings in the current study, teachers’ understanding of ADHD appear to be limited to the behaviours that are displayed by learners such as an inability to sit still, remain focused, complete work and sustain concentration. While teachers acknowledge the physiological and neurological basis of the disorder, they perceive emotional upset, poor diet, inadequate parenting and other barriers to learning as additional causal factors.Medication is viewed as the primary intervention strategy as it is considered to be fast acting and the most effective, this despite the environmental factors cited by the teachers as causal factors related to ADHD. A comparison of the results indicates no significant difference in private and public school teachers’ perceptions regarding the cause, interventions or incidence rate of ADHD. 

## Supplementary Files

Supplementary File 1
