# Supplementary material for: South African Foundation Phase Teachers’ Perceptions of ADHD at Private and Public Schools
Source: Int J Environ Res Public Health. 2015 Mar 11;12(3):3042–59. doi: 10.3390/ijerph120303042 (PMC4377951; doi:10.3390/ijerph120303042)
Supplement: Supplementary File 1 [file ijerph-12-03042-s001.pdf]

Supplementary Information

South African Foundation Phase Teachers’ Perceptions of ADHD at Private and Public Schools

UNIVERSITY OF THE WITWATERSRAND, JOHANNESBURG

UMTHOMBO WOPHUHLISO LOLUNTU
School of Human & Community Development

Psychology
School of Human & Community Development

Private Bag 3, Wits 2050, South Africa. Telephone: +27-11-717-4500/2/3/4. Fax: +27-11-717-4559

Foundation Phase Educators’ Perceptions of Attention Deficit Hyperactivity Disorder at Private and Public Schools

Questionnaire

Please answer the following questions by placing a tick in the appropriate box/column or by writing in the space provided.

Section A: Demographic Data

1. Gender:

Male	
Female	

2. Age Group:

20–25	
26–30	
31–35	
36–40	
41–45	
46–50	
Over 50	

3. Where did you qualify?

   Country:

   ____

   Institution:

   ____

4. What degree/diploma did you study for?

   ____

5. When did you graduate?

   ____

6. Number of years teaching experience:

1–5 years	
6–10 years	
11–15 years	
15– > years	

7. Have you completed any postgraduate courses?

Yes	
No	

   (**If yes**) Please elaborate.

   ____

   ____

8. Please specify the type of school at which you are currently teaching:

Inner City School (A school that is found in Johannesburg City Central or Braamfontein)	
Urban School (A school that is found in the suburbs surrounding Johannesburg City Central)	
Township School	
Other: ______________________________	

9. Which grade are you currently teaching?

Grade 0	
Grade 1	
Grade 2	
Grade 3	

10. How many learners are in your class?

Less than 15	
16–20	
21–25	
26–30	
31–35	
36–40	
41–45	
46–50	
More than 50	

11. Please specify the number of learners who fall into the following race groups in your class:

Black	
White	
Indian	
Coloured	
Other	

12. The following resources are available at my school:

Educational psychologist	
Occupational therapist	
Speech and Hearing therapist	
Clinical Psychologist	
Remedial therapist	

13. Have you attended any courses with regards to ADHD?

Yes	
No	

(If yes) Please elaborate.

____

____

____

____

Section B:

14. Do you have learners who have been medically diagnosed as having ADHD in your class?

Yes	
No	

(If Yes) How many? _________________

Specify the number of girls and boys:

Girls	
Boys	

What is their treatment plan?

Ritalin	
Concerta	
Behaviour modification	
Play Therapy	
Counselling	
Other	

Please specify. ____

Please specify which/all of these approaches to treatment are effective and why.

**Treatment plan**	**Effective**		**Reason**
	Yes	No	
Ritalin			____________ ____________
Concerta			____________ ____________
Behaviour Modification			____________ ____________
Play Therapy			____________ ____________
Counselling			____________ ____________
Other			____________

15. Do you have learners who you suspect of having ADHD in your class?

Yes	
No	

(If yes) How many? ________________

Specify the number of girls and boys:

Girls	
Boys	

16. What is your understanding of the term Attention Deficit Hyperactivity Disorder?

____
____
____
____

17. Children who have ADHD …

**Statement**	**Strongly Agree**	**Agree**	**Neither Agree nor Disagree**	**Disagree**	**Strongly Disagree**
Cannot sit still					
Daydream					
Fight with their classmates					
Do not complete work					
Cannot stay focused					
Do not listen					
Cannot start work					
Are easily distracted					
Are impulsive					
Are disruptive					
Talk constantly					
Are attention seeking					
Are fidgety					
Cannot concentrate					
Are disobedient					
Are emotionally sensitive					
Cannot follow instructions					
Cannot delay gratification					
Are disorganized					
Are forgetful					
Are noisy					
Shout out in class					
Other:_______________					

18. For a diagnosis of ADHD to be made …

**Statement**	**Strongly Agree**	**Agree**	**Neither Agree nor Disagree**	**Disagree**	**Strongly Disagree**
A child must be inattentive and/or hyperactive/impulsive					
The behaviour must have occurred before the age of seven years.					
The behaviour must be present in more than one setting (e.g., school and home).					
The behaviour must be present for more than six months.					

19. ADHD is caused by:

**Statement**	**Strongly Agree**	**Agree**	**Neither Agree nor Disagree**	**Disagree**	**Strongly Disagree**
Poor diet					
Brain dysfunction					
Depression					
Hereditary					
Lack of discipline in the home					
Auditory processing difficulties					
Visual processing difficulties					
Insecurity					
Lack of boundaries					
Too much television					
Reading difficulties					
Food additives, colourants and preservatives.					
Too much sugar					
Television games (including playstation, and x-box)					
Lack of physical activity					
Other: _______________					

20. In your opinion, a child suspected of suffering from ADHD should be assessed by a :

**Statement**	**Strongly Agree**	**Agree**	**Neither Agree nor Disagree**	**Disagree**	**Strongly Disagree**
Neurologist					
Pediatrician					
Educational Psychologist					

Occupational Therapist					
Speech and Hearing therapist					
General Practitioner					
Clinical Psychologist					
Other: _______________					

21. When I think a child may be suffering from ADHD I:

**Statement**	**Strongly Agree**	**Agree**	**Neither Agree nor Disagree**	**Disagree**	**Strongly Disagree**
Ask the parents to have an assessment done					
Move the child around the class					
Ask the parents to have a physical examination done					
Ask the parents to give the child Ritalin					
Put firm boundaries in place					
Discuss the child's diet with the parents					
Put the child at a desk of their own					
Try to limit distractions					
Put a behaviour modification plan in place (system of rewards)					
Other: _______________					

22. When referring to a specialist I expect the specialist to:

**Statement**	**Strongly Agree**	**Agree**	**Neither Agree nor Disagree**	**Disagree**	**Strongly Disagree**
Prescribe Ritalin					
Ask me to complete a Connors questionnaire					
Rule out physical causes of behaviour					
Offer me support/advice on how to deal with the child					

Please elaborate if there is anything else you would expect the specialist to do.

____

____

____

____

23. Please feel free to add anything else with regards to ADHD in the space provided.

____
____
____
____
____
____

Thank you for your time and cooperation.

© 2015 by the authors; licensee MDPI, Basel, Switzerland. This article is an open access article distributed under the terms and conditions of the Creative Commons Attribution license (http://creativecommons.org/licenses/by/4.0/).
